# Developing medical educators – a mixed method evaluation of a teaching education program

**DOI:** 10.3402/meo.v19.23868

**Published:** 2014-03-27

**Authors:** Marco Roos, Martina Kadmon, Michael Kirschfink, Eginhard Koch, Jana Jünger, Veronika Strittmatter-Haubold, Thorsten Steiner

**Affiliations:** 1 Institute of General Practice, University of Erlangen-Nuremberg, Erlangen, Germany; 2 Department of Surgery, Heidelberg University Hospital, Heidelberg, Germany; 3 Institute of Immunology, Heidelberg University Hospital, Heidelberg, Germany; 4 Department of Child and Adolescent Psychiatry, Heidelberg University Hospital, Heidelberg, Germany; 5 Department of General Internal Medicine and Psychosomatic, Heidelberg University Hospital, Heidelberg, Germany; 6 Academy of Adult Education, Heidelberg University of Education, Heidelberg, Germany; 7 Department of Neurology, Klinikum Frankfurt Höchst, Frankfurt, Germany

**Keywords:** faculty development, medical education, medical teacher, educational theory, collaborative feedback

## Abstract

**Background:**

It is well accepted that medical faculty teaching staff require an understanding of educational theory and pedagogical methods for effective medical teaching. The purpose of this study was to evaluate the effectiveness of a 5-day teaching education program.

**Methods:**

An open prospective interventional study using quantitative and qualitative instruments was performed, covering all four levels of the Kirkpatrick model: Evaluation of 1) ‘Reaction’ on a professional and emotional level using standardized questionnaires; 2) ‘Learning’ applying a multiple choice test; 3) ‘Behavior’ by self-, peer-, and expert assessment of teaching sessions with semistructured interviews; and 4) ‘Results’ from student evaluations.

**Results:**

Our data indicate the success of the educational intervention at all observed levels. 1) Reaction: The participants showed a high acceptance of the instructional content. 2) Learning: There was a significant increase in knowledge (*P*<0.001) as deduced from a pre-post multiple-choice questionnaire, which was retained at 6 months (*P*<0.001). 3) Behavior: Peer-, self-, and expert-assessment indicated a transfer of learning into teaching performance. Semistructured interviews reflected a higher level of professionalism in medical teaching by the participants. 4) Results: Teaching performance ratings improved in students’ evaluations.

**Conclusions:**

Our results demonstrate the success of a 5-day education program in embedding knowledge and skills to improve performance of medical educators. This multimethodological approach, using both qualitative and quantitative measures, may serve as a model to evaluate effectiveness of comparable interventions in other settings.

The drive for continuous improvement in medical education is propelled by both advancements in educational theory and research evidence, which is subsequently changing the traditional requirements of a medical educator (1–4). Hence, many medical faculties have endorsed development programs to improve teaching skills of their staff (5–7). A variety of different approaches have been surveyed, however, establishing the effectiveness of new faculty development programs and their impact on student education remains a challenge (8–10). In a systematic review by Steinert et al., the effects of faculty development interventions on knowledge, attitudes, and skills of educators on quality of education delivered, and on the institutions in which they worked, was reported (10). This review identified that repetitive interventions over time, using a deliberate adoption of theory of learning and educational principles, and the support of reflection and learning among participants, was effective. It was recommended that such interventions should be accompanied by process and outcome-oriented research, using multiple methods (quantitative as well as qualitative) in a performance-based way to assess changes on the basis of a conceptual framework (10). Kirkpatrick's model developed for measuring training effectiveness is one such useful framework (11). It measures effectiveness on four outcome levels: 1) the participants’ affective responses to training content and environment, 2) the impact of the training itself (level of learning), 3) the long-term outcome in job-related performance (level of behavior), and ultimately 4) institutional changes (level of results) (4, 5, 12–14).

The Heidelberg Medical Faculty implemented a 5-day education program in 2001 to support the implementation of its new medical curriculum HeiCuMed (Heidelberger Curriculum Medicinale) (15–17). The program prepares members of the medical faculty for their role as educators. Prior to 2001 within the old curriculum, the role of faculty members that were involved in teaching medical students was that of a directive instructor rather than that of a facilitator to support the learning process of students, as it is within the new curriculum. The 5-day education program targeted the entire teaching staff of the faculty (including those involved in both core sciences and clinical teaching). To date, over 1,000 faculty members have passed through this education program. It is delivered by qualified faculty members holding a Master degree in Medical Education or a Federal Certificate in Higher Education (in collaboration with external experts for adult education from University of Education, Heidelberg).

The content of the education program covers five essential objectives: 1) learning theory and educational principles, the ‘sandwich-architecture,’ and ‘constructive alignment’ as a general framework for teaching sessions, 2) simulation and skills labs as teaching methods and environments, 3) problem-based learning (PBL), 4) modern teaching assessment methods, and 5) reflection on the role of a medical educator. Each day of the course was concluded with a session of peer-coaching on an individual teaching session (facilitator–learner feedback as well as learner–learner feedback). The intent was to actively support participants to improve the ways in which they structured their teaching sessions as well as to enhance their teaching skills. These sessions were also designed to incorporate aspects of constructive alignment (18). The program content of each day is summarized in Table 1.

**Table 1:** Content of 5-day education program

	Day 1	Day 2	Day 3	Day 4	Day 5
Content	Educational principles - Professionalism as an educator? (S1.1) - Educational principles and theory of learning (S1.2) - The sandwich principle as a general architecture of learning sessions (S1.3)	Skills training - Use of simulation and skills lab (S2.1) - Doctor–patient communication training (Medi-KIT) (S2.2.) - Micro-teaching (video-supported presentations training) (S2.3)	Problem-based learning - General principles (S3.1) - Role as a tutor (S3.2) - Difficult situations in a tutorial (S3.3)	Assessment - Principles of assessment (miller pyramid) - MCQ (S4.1) - OSCE (S4.2)	Reflection on the role as a teacher (S5.2)

Peer-coaching sessions

(feedback in learning-session architecture, methodology, reflection on the role as a teacher) (S5.1)

The purpose of the present study was to evaluate the effectiveness of this 5-day education program applying Kirkpatricks’ framework. The questions we sought to answer were whether such an intervention improved the knowledge of the trainees on learning theory and educational principles, led to a better performance in teaching sessions, and improved peer-feedback. Interviews were conducted to evaluate the impact of the 5-day program with respect to changes in teachers’ behaviors and attitudes.

## Methods

### Study design

**Fig. 1:**
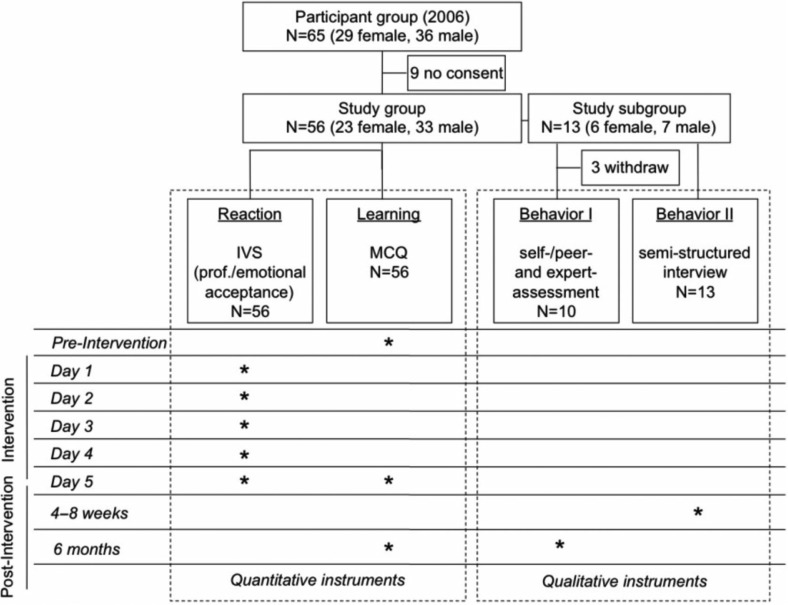
Study design and protocol; outline of applied instrument, ^*^ time points of measurement; IVS=Interactive Voting System.

To examine the effectiveness of the 5-day program, we adopted Kirkpatrick's model, applying quantitative and qualitative instruments, whose endpoints were defined on each of the four levels of Kirkpatrick's model. The study design (Fig. 1) was developed in compliance with the Helsinki Declaration for Ethical Principles for Medical Research Involving Human Subjects (www.wma.net). In accordance with the general regulation of the ethics committee at the Medical Faculty at University of Heidelberg, no ethic approval was needed. The study protocol did not include data of patients or medical intervention. Data were out of a quality management intervention of a faculty development program. Furthermore, data were collected anonymously (quantitative part), negative aftereffects for participants were not given and participation was on voluntary basis with an oral consent.

#### Reaction

The level of reaction is defined as the grade of acceptance of training content and environment and, therefore, has a direct impact on learning performance. We measured the level of reaction daily with a standardized questionnaire both on a professional and an emotional level: on the professional level, participants evaluated relevance for on-the-job performance (as a medical educator) for each session during the day on a 5-point Likert-scale (1=strongly agree to 5=strongly disagree); on the emotional level, participants evaluated the working atmosphere (1=very good to 5=poor) during the day.

#### Learning

A multiple choice questionnaire (MCQ) was developed consisting of 35 questions related to content of the 5-day program (objectives), set out in different question formats (type A, long-menu, and case-based questions) to measure basic knowledge and application of knowledge as well as analytical and synthesizing skills. Questions were developed by two educationalists and reviewed by an expert panel using the Delphi method (19). Participants answered the MCQ immediately before prior to starting the 5-day program, immediately after finishing the 5-day program, and 6 months later. Results are reported in sum scores.

#### Behavior

Qualitative instruments were applied to evaluate behavior. Behavior was defined as transfer of training content into teaching sessions (on-the-job performance) after completing the education intervention. Teaching sessions were observed by a peer (participant in program) and by an expert (facilitator of the 5-day program). In total, three formative assessments were conducted to evaluate the level of behavior: 1) self-assessment (participant in the program), 2) peer-assessment (by another participant in the program), and 3) expert-assessment (by a facilitator of the 5-day program). A standardized evaluation form was used to maximize objectiveness for each of the three assessments. The evaluation form provided assessors with different headings such as educator attitude toward teaching, application of educational principles, and coherence with learning objectives, as well as student–educator interaction. Assessors were requested to give feedback for each heading. All formative assessments were collected and processed as qualitative data on the basis of grounded theory.

In addition, semistructured interviews were conducted 4–8 weeks after the completion of the 5-day program to gain insights into changes in behavior and attitudes of participants, who were encouraged to reflect on their role as a medical educator. The interviews were guided by four questions: 1) Did the training program give you any incentives to improve your teaching sessions?, 2) Did you experience any changes in your personal view as a medical educator?, 3) Do you observe any obstacles in your work environment preventing you from being an effective medical educator?, and 4) What are your personal goals concerning effective medical education? All interviews were transcribed and processed as qualitative data on the basis of grounded theory.

### Study population

The target population consisted of all participants in the 5-day education program in 2006 (*n*=65). All participants were asked for consent. Nine participants declined (Fig. 1). Fifty-six participants responded on level of reaction and learning. However, because of the complexity of the qualitative evaluation of effectiveness, we randomly selected a study subgroup of 13 out of the 56 participants. This component evaluated the level of behavior according to Kirkpatrick's model. This subgroup was also selected for the observed teaching session (including self-, peer-, and expert-assessment) and the semistructured interview as described above. Experts (facilitators of the 5-day program) accompanied this subgroup during the post-training period.

### Data analysis

Results of MCQs, presented as means±SD were analyzed using Wilcoxon signed-rank test to describe differences between measurements (level of significance defined as *p*<0.05). In addition, effect size, *r*, and Cohen’s *d*, were calculated. To identify matched pairs, we used an individual reproducible code for each questionnaire. All quantitative data were statistically analyzed with SPSS 18 (SPSS Inc., an IBM Company, Chicago, USA) and SAS 8 (SAS Institute Inc., Heidelberg, Germany).

All qualitative data (peer, self-, and expert-assessment, written semistructured interviews) were analyzed using grounded theory (20) and qualitative content analysis (21). All qualitative data were transcribed verbatim and afterwards coded word by word by two separate investigators. Categories and subcategories were generated and conceptually organized. An expert panel (including two coding investigators) compared the generated categories, came to consensus on final domains, and related the number of responses to each category in a table.

## Results

Participants (nearly 60% were males) represented a complete cross-section of clinical and basic sciences disciplines of the medical school (see Table 2). Results presented according to the Kirkpatrick model are as follows:

1. Reaction: Table 3 presents results of standardized questionnaires of each session on a professional and emotional level. The means of professional level ranged from 1.54 (±0.73) to 2.74 (±1.05). Means of emotional level ranged from 1.48 (±0.76) to 2.36 (±1.02).
2. Learning (MCQ): Pre-training level (mean MCQ pre=10.76±3.12) and post-training level (mean MCQ post=16.84±3.23) analysis revealed a significant increase of education specific knowledge (*p*<0.001) with an effect size *r*=0.91 (Cohen's *d*=4.51). The latter was maintained at a high level 6 months post-training (mean MCQ after 6 months=15.09±4.06, *p*<0.001, effect size *r*=0.75, Cohen's *d*=2.27). Nevertheless, we found a significant decline in results in MCQ between measurement after the 5-day program and at 6-month follow-up (*p*<0.001, effect size *r*=0.56, Cohen's *d*=1.35).
3. Behavior: Transfer of theoretical content into teaching sessions (on-the-job performance): We evaluated 10 lectures (three participants of the study subgroup withdrew, because they could not perform their teaching sessions within the study period). We analyzed 177 text units from the self-, peer-, and expert-assessment. One hundred and twenty two (69%) text units were related to transfer of theoretical content into teaching sessions and 55 (31%) text units were individual formative feedback to the participant (peer coaching). Text units relating to the transfer of content were summarized in nine categories (Table 4). Qualitative analysis of formative feedback of peer coaching led to seven categories, divided in different groups (peer-, self-, and expert-assessment). Table 4 shows the distribution of different categories mentioned by each group.

**Table 2:** Description of study group

	*N*	%
Study group	56	100
Gender		
Females	23	41.1
Males	33	58.9
Discipline		
Basic sciences (e.g., biochemistry, physiology, pathology)	13	23.2
Core disciplines	23	41.1
(e.g., internal/family medicine, psychology)		
Surgical disciplines (e.g., visceral surgery, heart surgery, urology)	12	21.4
Dentistry	8	14.3
Study subgroup	13	100
Gender		
Females	6	46.2
Males	7	53.8
Discipline		
Basic sciences	3	23.1
Core disciplines	6	46.2
Surgical disciplines	3	23.1
Dentistry	1	7.7

**Table 3:** Results of evaluation of the theoretical content (level of reaction)

		Education session	Mean	SD
Day 1	S1.1	Professionalism as an educator	2.74	1.04
	S1.2	Educational principles and theory of learning	2.05	0.93
	S1.3	The sandwich principle as a general architecture of teaching/learning sessions	2.37	1.08
	A1	Atmosphere	1.93	0.78
Day 2	S2.1	Use of simulation and skills lab	1.80	0.98
	S2.2	Doctor–patient communication training (Medi-KIT)	1.80	0.84
	S2.3	Micro-teaching (video-supported presentation training)	1.54	0.73
	A2	Atmosphere	1.48	0.76
Day 3	S3.1	PBL-general principles	2.04	0.86
	S3.1	PBL-role as a tutor	2.18	0.98
	S3.3	PBL-Difficult situations in a tutorial	1.94	0.99
	A3	Atmosphere	1.76	0.86
Day 4	S4.1	Assessment-MCQ	1.71	0.86
	S4.2	Assessment-OSCE	2.00	0.80
	A4	Atmosphere	1.61	0.64
Day 5	S5.1	Peer coaching sessions	2.00	0.76
	S5.2	Reflections on the role as a teacher	2.73	1.05
	A5	Atmosphere	2.36	1.02

Quantitative assessment of reaction of participants to theoretical content and environment. S1.1–S5.2: measurements of professional acceptance, relevance for daily life of different sessions. A1–A5: measurements of acceptance on an emotional level (daily atmosphere). Means with SD (1=strongly agree/very good – 5=strongly disagree/poor).

**Table 4:** Qualitative peer-/expert-/self-assessment

Categories referring to evaluation of performance ‘Samples’	Peer-assessment, *n* (%)	Expert-assessment, *n* (%)	Self-assessment, *n* (%)
Using a sandwich structure in the teaching session ‘There was a transparent didactic architecture in the lecture (sandwich structure)’ ‘The sandwich architecture was realized very well’	8 (6.6)	8 (6.6)	
Transparency on learning objectives ‘Learning objectives were shown in the beginning …’ ‘All learning objectives were transparent to students’.	6 (4.9)	6 (4.9)	
Using an agenda as a (pre-)structure ‘An agenda was visual all the time’. ‘Good explanation of the agenda in the beginning’	7 (5.7)	7 (5.7)	
A central learning objective is recognizable ‘The central learning objective was obvious all the time’.	7 (5.7)	7 (5.7)	
A summary was shown to the students ‘Just in the right moment, there was a recapitulation’ ‘The end was used for a good recapitulation’	7 (5.7)	8 (6.6)	
Using/changing educational principles ‘The lecture included lots of learned educational principles’ ‘He used more educational principles’	6 (4.9)	7 (5.7)	
Transfer of learning content is supported ‘In a group work students had to transfer contents to different situations’. ‘All contents were shown in clinical cases’.	7 (5.7)	7 (5.7)	
Time management ‘The time management fits’.	5 (4.1)	5 (4.1)	
Interaction with students ‘By asking lots of questions, students interact a lot’ ‘There were many discussions … included’	7 (5.7)	7 (5.7)	
Total text units 122	60 (49.2)	62 (50.8)	
Categories referring to suggestions for further learning sessions			
Re-evaluation of time management	4 (7.3)	3 (5.5)	6 (10.9)
Facilitating group work	5 (9.1)	3 (5.5)	5 (9.1)
Using an agenda	2 (3.6)	3 (5.5)	5 (9.1)
Support interaction between students	3 (5.5)	0	3 (5.5)
Transparency on learning objectives	1 (1.8)	2 (3.6)	2 (3.6)
Using an ‘advance organizer’	0	2 (3.6)	2 (3.6)
Using an improved architecture (sandwich principle)	1 (1.8)	1 (1.8)	2 (3.6)
Total text units 55	16 (29.1)	14 (25.5)	25 (45.4)

Extracted categories of 122 text units in standardized evaluation forms of performance and 55 text units of suggestions for further learning are shown (gray column; no self-assessment).

In the semistructured interviews 157 text units were identified. One hundred and forty nine text units were related to the following four categories: 1) better knowledge on learning theory and educational principles, 2) reflection on the role as a medical educator, 3) barriers to being a good medical educator, and 4) goals of ‘good’ medical education (Table 5). Eight single text units could not be related to a definite category and were excluded from the analysis.

**Table 5:** Qualitative assessment of self-reflection (semistructured written interviews)

Categories ‘Samples’	Text units, *n* (%)
Better knowledge on learning theory and educational principles ‘The program gave me more possibilities to the architecture of teaching sessions’. ‘Understanding that usage of educational principles can improve learning impact of students’. ‘Feedback on my performance during the program improved my (preparation of) teaching sessions’. ‘In the past teaching as much facts as possible was important, … now using methods to support individual learning is in the foreground’.	41 (27.5)
Reflection on the role as a medical educator ‘Now I have a different view of quality in medical teaching’. ‘I feel more secure in my role as a medical educator’. ‘Since the program, I feel more engaged to reflect my responsibility as a medical educator’.	45 (30.2)
Barriers in daily life to be a good medical educator ‘In the clinical routine sometimes there is a loss of time to prepare my teaching lessons’. ‘Many times I feel unversed in using the “new” methods, but I hope it will be better with more practice’. ‘I think some didactic methods are artificial and not useful in daily teaching’.	25 (16.8)
Goals of improved medical education ‘Teach students in accordance with best practice’. ‘… improvement on medical knowledge, development of social and ethics competencies’. ‘To be an enthusiastic role model for students and to motivate students to prepare for a complex profession’. ‘A good physician is not a specialized “idiot” but an interdisciplinary team worker’.	38 (25.5)
Total text units	149 (100)

Table shows categories and samples of 149 text units reported in the semistructured interview.

## Conclusions

The primary goal of this study was to identify several factors that influence the developmental process of participants in the 5-day education program to become better medical educators. It found a high acceptance of the theoretical content, which was reflected in its approval on a professional and emotional level. This was an important prerequisite for achieving new knowledge and its transfer to on-the-job performance (in clinical settings), which was confirmed in the results of MCQ and qualitative measurements. Finally, it found a high concordance of self-, peer-, and expert-assessment in following clinical settings. According to the results of our study, the 5-day education program fulfills criteria from Kirkpatrick's framework on all explored levels measuring effectiveness of an education intervention.

As a precondition of achieving knowledge and of changing behavior a high satisfaction with the education structure and the content is needed (11). Our results confirmed satisfaction as shown in daily evaluations during the training, using items on professional aspects of the training content and on emotional reactions of our participants (Kirkpatrick's level of reaction). At the same time, education specific knowledge increased significantly (with high levels of effect size) during the training period and was maintained at the 6-month follow-up, as shown by the MCQ at three time points (*p*<0.001) (Kirkpatrick's level of learning). These findings are in line with previously published reviews and confirm the importance of an integrative theory of education motivation (2, 4, 12, 22). They also support the fact that reactions to education interventions have a fundamental impact on the engagement with theoretical content (knowledge and skill acquisition) and the transfer of new knowledge to job-performance (22).

A supportive organizational environment is indispensable for the sustainability of educational interventions to improve the transfer of new knowledge to job-performance and to stimulate changes in behavior as a medical educator (5, 23–25). This study identified three main supportive elements in the environment of our 5-day training program. First was establishing peer-coaching structures by implementing small group work of 4–5 peer tandems (learner with learner) with the aim of transferring the theoretical content into participants’ teaching portfolio (on-the-job performance) and to enhance development by collaborative working behaviors. Furthermore, a non-judgmental and peer-coaching environment with a constructive, formative feedback promotes a supportive learning environment and keeps participants motivated (26, 27). As a result, participants used their new knowledge for peer observation in the post-intervention period by evaluating and discussing job-related performance in peer-assessment, including suggestions for improvement (formative feedback). Supporting a feedback culture among the participants was one of the major training aims to encourage collegiality and collaboration within and across disciplines. Supporting collaborative formative feedback seemed to be the most valuable factor in learning processes, promoting effectiveness of training intervention (28).

Second, the fact that we found high agreement in observations between peer- and expert-assessment implies that our participants acquired the ability to apply theoretical content. They were supported in the transfer process into their teaching practice with peer- and own-performance reflections, as an important stage in their professional development (2, 14). The participants gave feedback to each other on content and quality of teaching performance. This individual feedback was comparable with expert feedback in quantity and quality, although the evaluation form only provides headings to guide and standardize feedback.

Third, the delivery of the intervention by professional facilitators with medical and pedagogical backgrounds was an important prerequisite for the effectiveness of this education intervention. This seems to be an essential structural element to avoid barriers between educators and clinicians (5, 10, 14).

Although the results of the study met the goals of our study, several limitations still remain. The needs of our institution did not allow for a control group. Therefore, we cannot be sure that some of the outcomes we attribute to the 5-day program are not actually due to a selection bias. Participation in the 5-day program is a prerequisite for the postdoctoral qualification for some participants. Furthermore, the 5-day program is compulsory for all faculty teaching staff within the faculty development program.

A second limitation is the small size of our study group (the whole faculty has approximately 1,200 members). However, for the intervention group, we reached 85% participation for quantitative (Level 1 and 2 of Kirkpatrick's framework) and 20% for qualitative measures (Level 3 of Kirkpatrick's framework). This might be a contrast to other findings in literature (29).

Third, long-term outcomes were measured at a 6-month follow-up, and some may regard this as too short an evaluation interval. However, we could demonstrate that the immediate and 6-month outcomes were still significantly higher than pre-intervention scores.

In conclusion, our findings indicate that the participants of our 5-days education intervention achieved a higher level of educational proficiency. Our participants achieved the required cognitive development. They felt familiar with learning theory and expressed their intention to apply the learned educational principles. Indeed, results of self- and peer-assessment revealed a direct impact of theoretical content on their job-related performance. Furthermore, peer-assessment motivated the participants to collaboratively work on the improvement of their teaching performance. Finally, the participants identified more with the role as a medical educator. We are convinced that this education intervention supported self-reflection of medical educators in their professional environment, promoted collegiality and collaboration within and across traditional discipline boundaries, and exerted an important impact on an effective faculty development (5, 10, 14, 30, 31).

More than 10 years after the initial implementation of the 5-day program, implemented to develop teaching staff in line with the new medical curriculum HeiCuMed, the success of this continuous quality improvement is confirmed by various studies on student evaluations and satisfaction (32, 33).
